# A River of Drugs

**DOI:** 10.3390/jox13010001

**Published:** 2022-12-21

**Authors:** Luigi Campanella

**Affiliations:** Department of Chemistry, Sapienza University, Piazzale Aldo Moro 5, 00185 Rome, Italy; luigi.campanella@uniroma1.it

The risk from EC concerns the abuse, not use, of drugs and, consequently, the excessive disposal of their metabolites. Hormones, antibiotics, anti-inflammatories and many others consumed beyond their requirements have a final destination in the waters of European rivers, from the Seine to the Thames, from the Danube to the Italian Po. This process, for many decades during last century, has not resulted in significant damages since the resulting concentrations did not exceed the nanograms/L scale; unfortunately, they have since grown during last 40 years to became much more dangerous, reaching about 0.01 ppm and more. This increase can be attributed, on one side, to the ease of prescription, leading to the high consumption of drugs, and on the other side, to the sometimes abundant content of the commercial confections, for instance, 20 pills when 10 should be enough. However, the factor probably the most responsible is the not always adequate functioning of water treatment plants due to their scarce maintenance and inadequate removal technologies. Some research has investigated this discrepancy and revealed a removal efficiency not higher than 70%. Some recent official data confirm this: for instance, laxatives sold in European pharmacies during one year amounted to 504 tons, paracetamol reached 232 tons, acetylsalicylic acid 131 tons, and penicillin 54 tons. The total number of commercial drug products in all of Europe is close to 2000. Such high drug consumption makes the pharmaceutical industry one of the highest producing industries in the secondary sector. In Italy, the most sold drugs treat cardiovascular and gastrointestinal pathologies. Residues from these drugs are not only dangerous to the environment, but also to human health as they pass into the food chain, in turn becoming responsible for many pathologies. Many research programs have been dedicated by the EU to look for new removal technologies to be integrated inside water treatment plants, the most advanced being adsorption and photodegradation, by which removal efficiency grows up approximately to 90% or more. There is also the problem of new drugs, for which removal technologies are obviously not always available. However, these drugs could be dealt with through other approaches, such as: following the ethical degree of consumption of pharmaceutical products by citizens; that is, their presence in the surface waters is strictly dependent on the behavior of citizens. We can conclude that the problem has many different faces: technical, economic, cultural, and so on. The hope to save our rivers is based in the responsible consumption of citizens, on the creativity of scientists, and on the ethics of the pharmaceutical industries.

